# Disruption of the l‐DOPA Receptor *Gpr143/OA1*‐Gene in Mice Creates a Unique Mixed Psychosis‐Like Phenotype

**DOI:** 10.1002/npr2.70080

**Published:** 2026-01-09

**Authors:** Yoshio Goshima, Haruko Nakamura, Motokazu Koga, Junka Koyama, Yayoi Kimura, Maya N. Vasishth, Evan Y. Snyder, Masashi Asai, Kenta Sakai, Daiki Masukawa

**Affiliations:** ^1^ Department of Molecular Pharmacology and Neurobiology Yokohama City University Graduate School of Medicine Yokohama Japan; ^2^ Department of Kampo Pharmacy Yokohama University of Pharmacy Yokohama Japan; ^3^ Department of Neurology Yokohama City University Graduate School of Medicine Yokohama Japan; ^4^ Department of Anesthesiology Kanagawa Cancer Center Yokohama Japan; ^5^ Advanced Medical Research Center Yokohama City University Yokohama Japan; ^6^ Sanford Burnham Prebys (SBP) Medical Discovery Institute La Jolla California USA; ^7^ Biomedical Sciences Graduate Program University of California‐San Diego La Jolla California USA; ^8^ Environmental Health and Prevention Research Unit Yokohama University of Pharmacy Yokohama Japan

**Keywords:** behavior, GPR143, l‐DOPA, ocular albinism 1, schizophrenia

## Abstract

GPR143, originally identified as the gene product of ocular albinism 1 (OA1), a G protein‐coupled receptor (GPCR), can function as a receptor for l‐3,4‐dihydroxyphenylalanine (DOPA), a precursor of dopamine (DA). To examine the physiological and pathophysiological roles of GPR143, we analyzed the behavior of *Gpr143* gene‐deficient (*Gpr143* ^*−/y*^) mice. We performed comprehensive behavioral analyses including the prepulse inhibition test, sucrose preference test, light–dark exploration test, etc. and microarray analysis. *Gpr143* ^*−/y*^ mice displayed a mixed psychosis‐like phenotype: impaired prepulse inhibition (a characteristic of schizophrenia) combined with reward system aberrations, depression, and heightened aggression (characteristic of mood disorders). By microarray analysis, we identified 17 downregulated and 20 upregulated genes in the forebrain of *Gpr143* ^*−/y*^ mice, genes putatively involved in serotonergic and/or dopaminergic transmission. These findings suggest that GPR143 plays a role in mesolimbic and mesocortical functions underlying sensory gating, reward, social hierarchy, cognition, and emotional regulation. These data further suggest both a new animal model and a unique therapeutic focus for a heretofore difficult to study and treat mixed psychosis‐like condition.

## Abstract 2

We analyzed the behavior of *GPR143* gene‐deficient mice. *GPR143‐KO* mice displayed a mixed psychiatric phenotype. GPR143 may play a role in mesolimbic and mesocortical functions underlying sensory gating, reward, social hierarchy, cognition, and emotional regulation.

## Graphical abstract

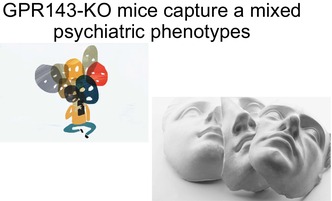

## 1 Introduction

l‐3,4‐dihydroxyphenylalanine (DOPA) has traditionally been considered solely a precursor of dopamine (DA). Since 1986, we have proposed that DOPA may itself also function as a neurotransmitter [1, 2, 3] Several lines of evidence suggest that G protein‐coupled receptor 143 (GPR143), originally identified as the gene product of ocular albinism 1 (OA1), an X‐linked type of albinism [4], acts as a receptor for DOPA [3, 5]. Since GPR143 is primarily and selectively expressed in retinal pigment epithelial cells [4] the non‐visual behavior of individuals affected with OA1 or mice with *Gpr143* deficiency has received little attention. However, GPR143 is also expressed, albeit at lower levels, in various brain regions, including the cortex, hippocampus, hypothalamus, substantia nigra, and ventral tegmental area [6]. In addition, multiple lines of evidence suggest that GPR143 plays a role in regulating DA D2 receptor (D2R) function [7, 8, 9]. In this study, we examined the non‐visual behavioral and gene expression consequences of *Gpr143‐*gene knockout and found that the gene‐deficient (*Gpr143* ^*−/y*^) mice exhibited a complex phenotype unlikely related to visual deficits, including behavior associated with schizophrenia (e.g., impaired prepulse inhibition (PPI)) yet also behavior associated with mood or affective disorders (e.g., depression; excessive reward responsiveness; heightened aggression and social dominance). Intriguingly, this mixed psychosis‐like phenotype, a complex difficult‐to‐categorize condition [10, 11] for which no specific genetic defect has been yet defined (although it can be familial), and few, if any, mouse models exist [11, 12]. Not only do our observations suggest that GPR143 may play a role in sensory gating, social behavior, reward, cognition, and emotional regulation—activities mediated by serotonergic and/or dopaminergic transmission in the mesolimbic and mesocortical systems—but they may also suggest a new animal model that can provide novel insights to study mixed psychosis‐like condition.

## 2 Materials and Methods

### 2.1 Mutant Mice and Experimental Design

*Wild‐type* (*Wt*) and *Gpr143* ^*−/y*^ mice were generated, and the genotypes of the offspring of mutant mice were assessed using polymerase chain reaction, as previously described [6]. Since the *Gpr143* gene is localized on the X chromosome, hemizygous male mice (*Gpr143* ^*−/y*^) and homozygous female mice (*Gpr143* ^*−/−*^) are both considered *Gpr143* knockout mice. Both *Gpr143* ^*−/y*^ and *Gpr143* ^*−/−*^ were viable, and no obvious gross defects were noted in these knockout mice [6]. A comprehensive behavioral test battery was conducted, recorded, and analyzed by highly trained blinded observers on single male mice that were at least 21 weeks of age. Female mice were excluded because their behavior is influenced by the estrous cycle. To minimize the effects of previous tests on subsequent tests, we performed the behavioral test battery in a specific order, with less stressful tests preceding more stressful ones, as described [13]. Each behavioral test was separated by at least 1 day to allow animals to recover. The order of the battery tests and the summary of each test result are provided in Table 1.

**TABLE 1:** Comprehensive behavioral test battery of *Gpr143* ^*−/y*^ mice compared to *Wt* mice.

Test	Measurement	Phenotype
Sensorimotor gating		
Startle reflex	Startle movement	↑
PPI	PPI	↑
Open field	Locomotor activity	→
Rota rod	Motor coordination	→
Beam	Grip strengthen	→
Cognitive system		
Cognition		
Objective recognition	Exploring time	→ or ↓ [34]
Sensory		
Habituation dehabituation	Smell sense	→
Valence		
Positive valence		
Two bottle choice	Sucrose preference	↑
Negative valence		
Light dark	Light/Dark transition	→
Contextual/Cued fear conditioning	Freezing time	→
Emotion system		
Elevated plus maze	Time on closed arm	→
Forced swim	Immobility duration	↑
Social process		
Social recognition	Exploring time	→
Tube	Number of “Wins” or “Loses”	↑

All mice were bred and maintained under standardized environmental conditions (temperature, humidity, and light–dark cycle) within the same facility. Although the extract littermate pairing could not be confirmed for all experiments, *Wt* and *Gpr143* ^*−/y*^ mice were derived from heterozygous crossings of the same colony and were age‐matched at the time of testing. We performed behavioral analysis of *Wt* and *Gpr143* ^*−/y*^ mice and evaluated concurrently. Mice were housed in a standard mouse facility and fed an autoclaved diet and water. All procedures were conducted in accordance with the guidelines of the Institutional Animal Care and Use Committee of the Yokohama City University Graduate School of Medicine (Approval No. F‐A‐14‐046). Throughout the experiment, all efforts were made to minimize the number of animals used and their suffering.

### 2.2 Behavioral Tests

Manual scoring of behavioral changes in mice was performed independently by two observers, who visually assessed and recorded the frequency and duration of specific behaviors. In cases where discrepancies occurred, the recorded videos were reviewed again for re‐analysis. The manual scoring paradigm and raw data are shown in Table S1.

#### 2.2.1 Pre‐Pulse Inhibition (PPI) Test

PPI refers to the phenomenon in which a response to a strong startling stimulus is weakened or inhibited when preceded by a weaker, non‐startling stimulus. Its loss in animals and patients is associated with a schizophrenic phenotype. A startle reflex measurement system (O'Hara & Co. Ltd.) was used to measure startle response and PPI as described [14]. The test session began by placing a mouse in a plastic cylinder and leaving it undisturbed for 10 min. White noise (40 ms) served as the startle stimulus for all trial types. The startle response was recorded for 140 ms starting from the onset of the prepulse stimulus. The background noise level in each chamber was 70 dB. The peak startle amplitude recorded during the 140‐ms sampling window was used as the dependent variable. The intensity of the startle stimulus was set at either 110 or 120 dB. The prepulse sound (74 or 78 dB) was presented 100 ms before the startle stimulus. Four combinations of prepulse and startle stimuli were used (74 and 110, 78 and 110, 74 and 120, and 78 and 120 dB). The trials were presented in six blocks, with each block containing one occurrence of each trial type in a pseudorandom order.

#### 2.2.2 Open Field Test

The open‐field test was conducted as previously described [15] to assess locomotor function and anxiety‐like behavior. Each subject was placed at the center of a test chamber (50 × 50 × 40 cm; O'Hara & Co. Ltd., Tokyo, Japan) under standard lighting conditions (70 lx). Activity in the open field was quantified using imaging software (O'Hara & Co. Ltd.). Travel distance (cm), total movement duration (s), and time spent in the center (s) were recorded.

#### 2.2.3 Rotarod Test

Motor coordination and balance were assessed using an accelerating rotarod (Muromachi Kikai Co. Ltd.) as previously described [16, 17]. Mice were placed on a cylinder that gradually accelerated from 4 to 40 rpm over a maximum duration of 300 s. Each mouse underwent three trials, and the latency to fall was recorded, with a maximum latency of 300 s.

#### 2.2.4 Balance Beam Test

Motor coordination and balance were also assessed by evaluating the mice's ability to traverse a graded series of narrow beams to reach an enclosed safety platform [14, 18]. The beams were placed horizontally 50 cm above the bench surface, with one end mounted on a narrow support and the other end attached to an enclosed box that served as a refuge for the mice. Overhead lights were positioned above and to one side of the beam's starting point. During training, mice were placed at the start of the square beam and trained for 1 day to traverse the beam towards the enclosed box. Following the training phase, each mouse completed a single trial on each square and round beam, progressing from the narrowest to the widest beam. Mice were given a maximum of 60 s to traverse each beam. The latency to traverse each beam and the number of hind feet slips were recorded for each trial.

#### 2.2.5 Olfactory Habituation/Dishabituation Test

To assess olfaction, mice underwent an olfactory habituation/dishabituation test [19]. The odorant stimuli were tap water, vanilla extract (Golden Kelly Pat. Flavor Co. Ltd., Osaka, Japan) diluted 1:100 in tap water, and bitter almond extract (Golden Kelly Pat. Flavor Co. Ltd.) diluted 1:100 in tap water. The selection of these odorants and dilutions was based on data from pilot experiments in previous studies [20]. The order of presentation was as follows: water (three times), vanilla (three times), and bitter almond (three times). An experimenter recorded the cumulative time each mouse spent sniffing the cotton‐tipped applicator using a stopwatch. Sniffing was defined as described previously [14].

#### 2.2.6 Sucrose Preference Test (Two‐Bottle Choice Test)

The sucrose preference test, a model for measuring stress‐induced anhedonia, is based on a two‐bottle choice paradigm [21, 22, 23]. The two‐bottle choice test has been used to measure nutrient preference. Each mouse was placed in a square aluminum cage and habituated for approximately 24 h. During this habituation period, the mouse had access to two bottles containing water. After habituation, one of the two bottles was replaced with a bottle containing 5% sucrose solution. The mouse was then given access to both the water and sucrose bottle, and the consumption was monitored for 24 h. Consumption was measured as grams of a liquid per gram of body weight. The ratio of sucrose solution intake relative to the total liquid intake (water + sucrose solution) was considered an indicator of taste preference.

#### 2.2.7 Light–Dark Exploration Test

The light–dark exploration test was conducted as previously described [24, 25, 26] to assess anxiety‐like behavior. The larger compartment was transparent, open‐topped, and brightly illuminated by a 40‐W desk lamp (1000 lx). The smaller compartment had a closed top and was painted black. Mice were individually placed in the center of the light compartment, facing away from the partition, and allowed to freely explore the apparatus for 10 min. The number of light–dark transitions between the two compartments and the total time spent in the dark compartment were automatically recorded using ANY‐maze software.

#### 2.2.8 Contextual and Cue Fear Conditioning Test

Each mouse was placed in a test chamber and allowed to explore freely for 2 min. A 55‐dB white noise, serving as the conditioned stimulus (CS), was presented for 30 s, followed by a mild (2 s, 0.35 mA) foot shock, which acted as the unconditioned stimulus (US). Two additional CS‐US pairings were presented with an interstimulus interval of 2 min. Context testing was conducted in the same chamber 1 day after conditioning. Cued testing in an altered context was performed in a triangular box made of white opaque Plexiglas, located in a different room, 1 day after conditioning.

Data acquisition, stimulus control (tones and shocks), and data analysis were performed automatically with Image FZ software (O'Hara & Co. Ltd.). Images were captured at a rate of 1 frame per second. For each pair of successive frames, the area (in pixels) corresponding to the movement of the mouse was measured. If this area was below a certain threshold (20 pixels), the behavior was judged as freezing. If the area equaled or exceeded the threshold, the behavior was considered nonfreezing. Freezing episodes lasting < 2 s, the defined time threshold, were excluded from analysis [14].

#### 2.2.9 Elevated Plus Maze Test

The elevated plus maze test was conducted as previously described to assess anxiety‐like behavior [27]. The apparatus consisted of two open arms and two closed arms that extended from a common central platform. A small, raised lip around the perimeter of the open arms prevented the mice from falling. The apparatus was made of polypropylene, with gray floor and gray walls, and was elevated 40 cm above the floor. Each mouse was individually placed in the center square, facing an open arm, and allowed to freely explore the apparatus under overhead fluorescent lighting (200 lx) for 5 min. Time spent in the open arms and the number of open and closed arm entries (defined as all four paws entering an arm) were recorded by a highly trained observer using behavioral scoring software (ANY‐maze; Muromachi Kikai Co. Ltd., Tokyo, Japan).

#### 2.2.10 Social Recognition Test

The social recognition test was conducted to assess cognitive function. Mice were habituated to the testing chamber for 15 min. For training, each mouse was placed in the chamber with a naïve mouse for 10 min. One hour later, the mouse was returned to the chamber and exposed to both the same (familiar) mouse and a novel mouse for 10 min. The time spent exploring each mouse was recorded during both the training and test phases.

#### 2.2.11 Confrontation Tube Test (Tube Test)

The confrontation tube test (tube test) is a validated measure of social hierarchy in mice [28, 29, 30]. We modified a previously described method [31] and used a transparent Plexiglas which is sufficient for a single adult mouse to pass through without reversing the direction. For training, each mouse was released at alternating ends of the tube and encouraged to traverse it, occasionally assisted by a plastic stick gently pushing from behind. Each animal underwent eight training trials per day for two consecutive days. Two mice were released simultaneously from opposite ends of the tube and allowed to meet in the middle. The mouse that retreated first from the tube within 2 min was designated the “loser” of that trial. If neither mouse retreated within 2 min, the trial was repeated. Between each trial, the tube was cleaned with 70% ethanol. In the control and knockout groups, paired encounters were staged while taking body weight into consideration.

#### 2.2.12 Forced Swim Test

The forced swim test was conducted as previously described [32]. Mice were placed in individual glass cylinders (height: 24.5 cm; diameter: 19 cm) filled with water (23°C–25°C) to a depth of 15 cm, ensuring that they could not support themselves by touching the bottom. Swim sessions lasted for 6 min, and the water was changed between subjects. All test sessions were recorded, and immobility duration was automatically quantified by ANY‐maze. Mice were considered immobile when making only the minimal movements necessary to keep their heads above water.

### 2.3 Image Analysis

The applications used for the behavioral studies (Image HA, Image EP, Image PS, Image FZ, and Image BM) were based on the NIH Image program (developed at the U.S. National Institutes of Health and available at http://rsb.info.nih.gov/ij). These applications were modified for each test by D. Masukawa. Image FZ is freely available at the following http://www.mouse‐phenotype.org/software.html.

### 2.4 Microarray Analysis

Total RNA was extracted from forebrain tissues using the RNeasy Mini kit (Qiagen, Hilden, Germany). The RNA integrity number (RIN) of all total RNA samples was assessed via Bioanalyzer electropherograms (Agilent technologies Japan Ltd., Tokyo, Japan). Samples with RIN values higher than 6.5 were included in the analysis. For each mouse group (*n* = 3), RNA samples were pooled equally. A total of 100 ng of RNA was processed for microarray analysis using the GeneChip WT PLUS Reagent Kit (Thermo Fisher Scientific Inc., Waltham, MA, USA) following the manufacturer's protocol. The resultant single‐strand cDNA was fragmented, biotin‐labeled, and hybridized to the GeneArray Mouse 2.0ST Array. The arrays were washed, stained, and scanned using the Affymetrix 450 Fluidics Station and GeneChip Scanner 3000 7G (Thermo Fisher Scientific Inc., Waltham, MA, USA). Expression values were generated using Expression Console software, version 1.3 (Thermo Fisher Scientific Inc.), with default robust multichip analysis parameters. These analyses were performed by Kurabo Industries Ltd. (Osaka, Japan). Raw and standardized (log‐transformed) microarray data were registered in the Gene Expression Omnibus (GEO) database at the National Center for Biotechnology Information (NCBI). GEO accession number was GSE157828. Signals from *Wt* and *Gpr143* ^*−/y*^ mice (*n* = 3) in the array data were compared using Student's *t*‐test. The volcano plot displays log_2_(fold change) on the *x*‐axis and –log_10_(raw *p*‐value) on the *y*‐axis.

### 2.5 Enrichment Analysis

To identify the downstream molecules, gene set enrichment analysis was performed using the EnrichR web server (https://maayanlab.cloud/Enrichr/) and the R package enrichR to query multiple gene‐set libraries from the ARCHS4 coexpression database. The result of pathway analysis displays –log_10_(raw *p*‐value) on the *y*‐axis.

### 2.6 Ingenuity Pathway Analysis

To perform functional annotation, significantly different genes (*p* < 0.05) between *Gpr143* ^*−/y*^ and *Wt* in microarray analysis were imported to the Ingenuity Pathway Analysis Tool (IPA Tool; Ingenuity Systems, Redwood City, CA, USA; http://www.ingenuity.com; Analysis Creation Date: 2025‐05‐12 Content version: 134816949, Release Date: 2025‐02‐18; Qiagen, Hilden, Germany) using Fisher's exact test (https://pubmed.ncbi.nlm.nih.gov/38475964/). Upstream regulator analysis was then performed. Upstream regulators that met the criteria of *p* < 0.05 and contained a minimum of three genes with expression changes are shown in Figure 5. The result of upstream regulator analysis displays –log_10_(raw *p*‐value) on the *y*‐axis.

### 2.7 Statistical Analysis

Statistical analysis was conducted using GraphPad Prism 10.1.1 (GraphPad, La Jolla, CA, USA). Data were analyzed using paired *t*‐test, one‐way ANOVA, or two‐way repeated measures ANOVA, unless stated otherwise. Values in graphs are expressed as mean ± SEM. *Post hoc* analysis was performed using Fisher's protected least significant difference (Fisher's PLSD) for multiple comparisons. Genotype × time or drug × time interactions were analyzed using repeated measures ANOVA. The specific statistical treatments for each dataset are described in the respective figure legends.

## 3 Results

### 3.1 Behavioral Analysis

The *Gpr143* ^*−/y*^ mice appeared healthy, and, other than having irregular hypopigmentation in their retinae, displayed no obvious differences from *Wt* mice in body weight and other physical characteristics. *Gpr143* ^*−/y*^ mice showed normal locomotor activity (as assessed by open field testing) (Figure S1), balance and motor coordination (as assessed by rotarod and balance beam tests) (Figure S2), and sensory function (as assessed by the hot plate test [33], odor function (Figure S3)).

Although we previously observed no difference between *Wt* and *Gpr143* ^*−/y*^ mice in their preferences for a novel object during a 30‐min retention period [34], during a 24‐h retention period, as performed here, *Gpr143* ^*−/y*^ mice spent less time exploring the novel object than *Wt* mice, suggesting a slight decline in cognitive function. The finding on the contextual and cue‐based fear conditioning test in the current study further suggests a minor, if any, decline in cognitive function in *Gpr143* ^*−/y*^ mice (Figure S3).

**FIGURE 1:**
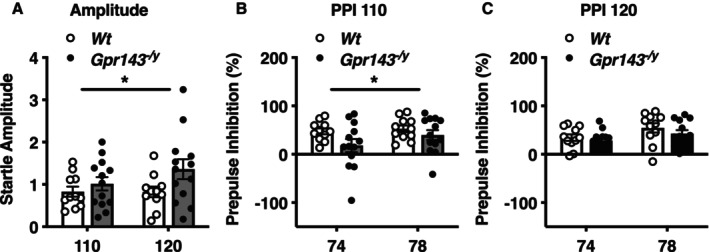
Impaired PPI in *Gpr143* ^*−/y*^ mice, a phenotype associated with schizophrenia. (A) Startle amplitude was higher in *Gpr143* ^*−/y*^ mice compared to *Wt* mice. (B) The percentage of PPI was significantly reduced in *Gpr143* ^*−/y*^ mice with a 110 dB‐startle stimulus. (C) No significant difference in PPI was observed with a 120 dB‐startle stimulus (Right). *p < 0.05. Statistical significance was determined using a two‐way ANOVA. Data are shown as mean ± SEM (*n* = 11–13).

To examine sensorimotor gating, we performed the PPI test. The startle response amplitudes were higher in *Gpr143* ^*−/y*^ mice than in *Wt* mice, and the percentage of PPI was reduced (Figure 1), indicating impaired sensorimotor gating, a phenotype associated with schizophrenia.

**FIGURE 2:**
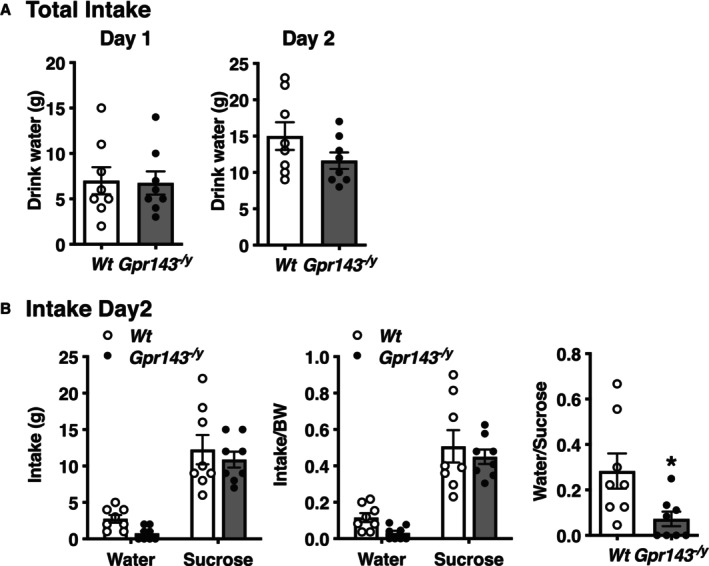
Increased sucrose preference in *Gpr143* ^*−/y*^ mice, a phenotype associated with an altered reward system. While decreased sucrose intake compare with wild type (Wt) is traditionally viewed as a surrogate for anhedonia, increased sucrose intake can be viewed as a sign of excessive reward‐seeking behavior & a precursor to addiction. (A) Total water intake did not differ between *Wt* and *Gpr143* ^*−/y*^ mice on either day 1 or day 2. (B) *Gpr143* ^*−/y*^ mice exhibited a significant preference to sucrose (5%) in a two‐bottle choice test. *p < 0.05. Statistical significance was determined using unpaired *t*‐test. Data are shown as mean ± SEM (*n* = 8).

The sucrose preference test (SPT) has long been used to probe the pathways that regulate reward processing and sensitivity, thus probing the underlying dopaminergic and serotonergic circuits. While decreased sucrose intake compared with that of Wt can be interpreted as a surrogate for anhedonia (the most common use of the SPT), increased sucrose intake compared to Wt can be interpreted as a sign of excessive reward‐seeking behavior, a precursor to addiction and a characteristic of bipolar disorder [22]. In the two‐bottle choice test between water and sucrose solutions, there was no difference in total water intake between *Wt* and *Gpr143* ^*−/y*^ mice. However, *Gpr143* ^*−/y*^ mice displayed a stronger preference for sucrose, despite no overall difference in total intake of water and sucrose solution (Figure 2), suggesting an alteration in reward‐associated behaviors. For example, the SPT changes observed in the *Gpr143* ^*−/y*^ mice were similar to the higher sucrose preference reported for mice receiving morphine [22].

To assess negative valence, we performed the light–dark exploration test. No significant difference was observed in time spent in the light or dark compartment, or the number of entries into the dark compartment between *Wt* and *Gpr143* ^*−/y*^ mice (Figure S4). We also performed the contextual and cue‐based fear conditioning test. *Gpr143* ^*−/y*^ mice did not show significant differences in freezing time during either the conditioning phase or the contextual/cue test (Figure S5).

**FIGURE 3:**
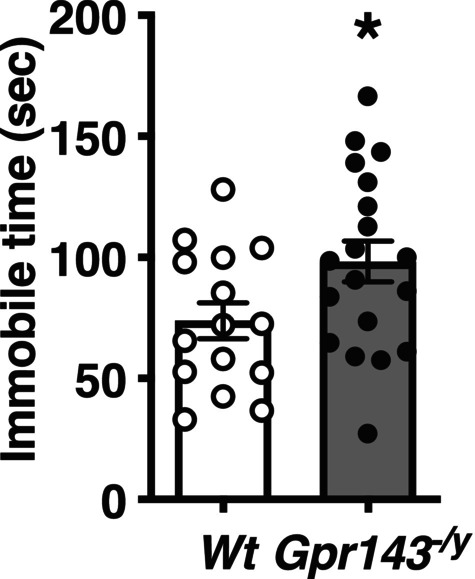
Depressive‐like behavior was increased in *Gpr143* ^*−/y*^ mice. *Gpr143* ^*−/y*^ mice exhibited a significantly longer total immobility time in the forced swim test compared to *Wt* mice, indicating depressive‐like behaviors. *p < 0.05. Statistical significance was determined using unpaired *t*‐test (*n* = 15–19).

To evaluate anxiety‐related behavior, we performed the elevated plus maze test. There was no significant difference in the time spent in open or closed arms, or in the distance traveled within them (Figure S6). To assess stress responsivity and behavioral despair, we conducted the forced swim test, which is also used to evaluate depression. In this test, *Gpr143* ^*−/y*^ mice exhibited increased immobility time compared to *Wt* mice, a sign of depression (Figure 3).

**FIGURE 4:**
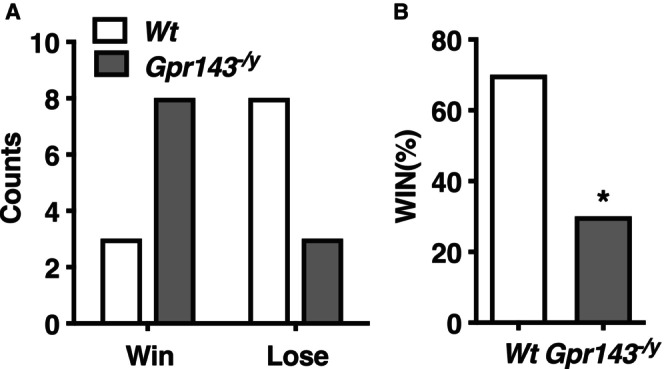
Enhanced social dominance in *Gpr143* ^*−/y*^ mice. (A) Number of “wins” or “loses” for *Wt* and *Gpr143* ^*−/y*^ mice. (B) Percentage of “wins” and “loses” of *Gpr143* ^*−/y*^ mice. *p < 0.05. Statistical significance was determined using a two‐way ANOVA (*n* = 11).

In the social interaction test, there was no significant difference in interaction time between *Wt* and *Gpr14* ^*3−/y*^ mice (Figure S7). In contrast, in the tube test of social dominance, the percentage of trials recorded as a ‘win’ was higher in *Gpr143* ^*−/y*^ mice compared to *Wt* mice (Figure 4).

Overall, these results suggest that *Gpr143* ^*−/y*^ mice exhibit a mix of sensorimotor gating and minor cognitive abnormalities, consistent with schizophrenia, and increased reward‐seeking, aggressive, and anxiety‐related behaviors (consistent with a mood or affective disorder) (Table 1).

### 3.2 Microarray Analysis

**FIGURE 5:**
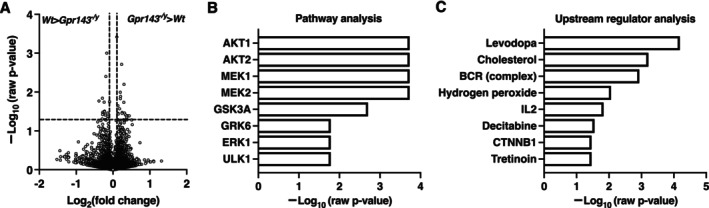
Differential gene expression in *Gpr143* ^*−/y*^ mice. (A) Volcano plot showing differential gene expression between *Gpr143* ^*−/y*^ and *Wt* mice. The plot displays log_2_(fold change) on the *x*‐axis and –log_10_(raw *p*‐value) on the *y*‐axis. (B) Pathway analysis was performed by using the ARCHS4 coexpression database in EnrichR. (C) Upstream regulators predicted by the IPA tool. The analysis displays –log_10_(raw *p*‐value) on the *y*‐axis (B, C). Target molecules in the dataset are shown in Tables 3 and 4.

Given that psychosis‐like disorder has not yet been associated with specific genetic abnormalities (beyond a tenuous one with 22q11.2 deletion [35]), to begin elucidating the genetic underpinnings that might contribute to the abnormal behaviors seen in the *Gpr143* ^*−/y*^ mice, we conducted a microarray analysis. We identified significant (*p* < 0.05) upregulation and downregulation of several mRNAs in *Gpr143* ^*−/y*^ mice compared to *Wt* mice (Figure 5A and Table 2). To explore the signaling pathways associated with GPR143 deficiency, we performed enrichment and downstream analysis. Using differentially expressed genes, enrichment analysis with the ARCHS4 database in EnrichR revealed significant enrichment of signaling pathways downstream of GPR143, including AKT1/2, MEK1/2, GSK3A, ERK1, GRK6, and ULK1 (Figure 5B and Table 3). Most of these signaling molecules and pathways have been implicated in the pathophysiology of schizophrenia and bipolar disorder. For example, a decrease in AKT1 protein levels and levels of phosphorylation of GSK3β at Ser9 in the peripheral lymphocytes and brains of individuals with schizophrenia was reported [36]. Akt‐mammalian target of rapamycin (mTOR) signaling was reduced in the prefrontal cortex in a subset of bipolar disorder subjects [37]. Exosome sequence analysis and genotyping suggested that rare ULK1 variants were involved in susceptibility to schizophrenia [38]. Complementary IPA upstream analysis suggested that DOPA is the most significant upstream regulator influencing these gene expression changes (Figure 5C and Table 4).

**TABLE 2:** Differential gene expression in wild type (*Wt*) and Gpr143 gene‐deficient (*Gpr143* ^*−/y*^ *)* mice (*n* = 3).

Gene	Gene name	Raw fold‐change	Raw *p*‐value
Wt > Gpr143^−/y^			
Gm25198	Predicted gene, 25198 [gene_biotype:miRNA transcript_biotype:miRNA]	0.77	0.017
Gm23510	Predicted gene, 23510 [gene_biotype:snRNA transcript_biotype:snRNA]	0.82	0.049
A830018L16Rik	A830018L16 gene (A830018L16Rik), transcript variant 1	0.82	0.014
Snord42a	C/D box 42A	0.82	0.038
Ndufb3	NADH dehydrogenase (ubiquinone) 1 beta subcomplex 3	0.82	0.033
Plekha5	Pleckstrin homology domain containing, family A member 5	0.83	0.036
Abl2	V‐abl Abelson murine leukemia viral oncogene 2, transcript variant 1	0.85	0.018
Tnks	Tankyrase, TRF1‐interacting ankyrin‐related ADP‐ribose polymerase	0.85	0.031
Pik3c2b	Phosphoinositide‐3‐kinase, class 2, beta polypeptide	0.85	0.032
Gm3095	Predicted gene 3095, transcript variant X1	0.86	0.039
Vav2	Vav 2 oncogene	0.87	0.004
Dcun1d5	DCN1, defective in cullin neddylation 1, domain containing 5	0.89	0.001
Vps13d	Vacuolar protein sorting 13 D, transcript variant 2	0.90	0.049
Nifk	Nucleolar protein interacting with the FHA domain of MKI67	0.91	0.048
Nr2f1	Nuclear receptor subfamily 2, group F, member 1	0.91	0.035
Osbpl9	Oxysterol binding protein‐like 9, transcript variant c	0.91	0.043
Eif5b	Eukaryotic translation initiation factor 5B	0.94	0.018
Wt < Gpr143^−/y^			
Ctcf	CCCTC‐binding factor	1.07	0.000
Capn10	Calpain 10 (Capn10)	1.10	0.022
Wdr1	WD repeat domain 1	1.10	0.035
Sh3bp5l	SH3 binding domain protein 5 like, transcript variant 1	1.10	0.037
Laptm5	Lysosomal‐associated protein transmembrane 5	1.13	0.027
Rpl31	Ribosomal protein L31, transcript variant 1	1.15	0.010
Klf16	Kruppel‐like factor 16	1.15	0.015
Olfml2b	Olfactomedin‐like 2B	1.15	0.013
Aldh9a1	Aldehyde dehydrogenase 9, subfamily A1	1.18	0.002
Klhl17	Kelch‐like 17	1.18	0.035
Imp4	IMP4, U3 small nucleolar ribonucleoprotein, homolog	1.19	0.034
Copz2	Coatomer protein complex, subunit zeta 2	1.22	0.018
Hes6	Hairy and enhancer of split 6	1.23	0.006
Tmem254b	Transmembrane protein 254b, transcript variant 1	1.24	0.035
Ccbl2	Cysteine conjugate‐beta lyase 2, transcript variant 1	1.26	0.034
Nhlrc3	NHL repeat containing 3	1.26	0.039
Cyp2j6	Cytochrome P450, family 2, subfamily j, polypeptide 6	1.29	0.042
Hdgf	Hepatoma‐derived growth factor	1.29	0.047
Hbegf	Heparin‐binding EGF‐like growth factor	1.32	0.041
Anapc2	Anaphase promoting complex subunit 2	1.36	0.041

*Note:* The other details are shown in Supporting Information.

**TABLE 3:** Differentially expressed genes in pathway analysis from EnrichR.

Pathway	Raw *p*‐value	Target molecules in dataset
AKT1	0.000186	SH3BP5L, WDR1, HDGF, KLF16, ANAPC2
AKT2	0.000186	SH3BP5L, KLHL17, HDGF, KLF16, ANAPC2
MEK1	0.000186	SH3BP5L, WDR1, HDGF, KLF16, ANAPC2
MEK2	0.000186	SH3BP5L, WDR1, HDGF, KLF16, ANAPC3
GSK3A	0.00198	SH3BP5L, HDGF, KLF16, ANAPC2
GRK6	0.0164	SH3BP5L, LAPTM5, ANAPC2
ERK1	0.0164	SH3BP5L, KLF16, ANAPC2
ULK1	0.0164	SH3BP5L, KLF16, ANAPC2

**TABLE 4:** Differentially expressed genes in upstream analysis from IPA analysis.

Upstream regulator	Raw *p*‐value	Target molecules in dataset
DOPA	0.0000657	CAPN10, HBEGF, KLF16, LAPTM5, WDR1
Cholesterol	0.000611	HBEGF, HES6, LAPTM5
BCR (complex)	0.00117	CTCF, IMP4, LAPTM5
Hydrogen peroxide	0.00869	CTCF, HBEGF, RPL31
IL2	0.0148	CTCF, HBEGF, LAPTM5
Decitabine	0.0281	ANAPC2, HBEGF, HDGF
CTNNB1	0.0345	HBEGF, HDGF, RPL31
Tretinoin	0.0346	ALDH9A1, CTCF, HBEGF, HDGF

## 4 Discussion

Here, following a comprehensive behavioral analysis of the *Gpr143* ^*−/y*^ mouse, we observe it to display a mixture of psychosis‐like phenotypes that cut across those used to model disorders like schizophrenia as well as those used to model affective or mood disorders like bipolar disorder.

A striking finding is that the behavior we report now for the *Gpr143* ^*−/y*^ mice was a positive result of PPI and preference for sucrose, reminiscent of a common phenotype associated with animal models of schizophrenia (Figures 1 and 2) [39]. It appears to contradict our previous findings that *Gpr143* ^*−/y*^ mice exhibit an impairment of D2R function [7, 8]. *GPR143* ^*−/y*^ mice showed a lowered behavioral response to a D2R agonist quinpirole, and to acute and chronic treatment with nicotine and methylphenidate [40, 41]. We confirmed that there was no difference between wild‐type and *Gpr143* ^*−/y*^ mice in the release of DOPA and DA monitored by striatal microdialysis [7, 41]. The immunohistochemical staining with anti‐TH antibody in the brain in *Gpr143* ^*−/y*^ mice was comparable to that in *Wt* mice [7]. Although the reasons behind these conflicting results remain unclear, it could be due to compensatory mechanisms arising from the deficiency of the *Gpr143* gene. In addition, PPI is associated with multiple neural circuits and neurotransmitters including glutamate, acetylcholine, DA, and serotonin. Thus, it is also possible that the enhanced PPI may be caused by alteration of other neurotransmitter systems in the brain. Interestingly, it is becoming increasingly recognized that such non‐dopaminergic systems are implicated in various aspects of schizophrenia, including affective lability, positive, negative, and cognitive symptoms.

In the novel object recognition test with a retention period of 24 h, *Gpr143* ^*−/y*^ mice spent less time exploring the novel object compared to *Wt* mice, suggesting a slight decline in cognitive function in place recognition [34].

In the forced swim test, *Gpr143* ^*−/y*^ mice became immobile more rapidly and for longer than *Wt* mice, indicating a greater degree of behavioral despair and depression than *Wt* mice, suggestive of a mood or affective disorder.

The SPT is typically used to evaluate the motivation to obtain a pleasurable substance, like a sucrose solution, over water [22, 23, 42]. In other words, it has long been used to probe the pathways that regulate reward processing and responsiveness, typically the mesolimbic dopaminergic pathways involving the ventral tegmental area and the nucleus accumbens, as well as serotonergic pathways. As noted previously, while decreased sucrose intake compared with that of Wt is viewed as a surrogate for anhedonia (the most common use of the SPT), increased sucrose intake compared to Wt has been used as a sign of excessive reward‐seeking behavior, a precursor to addiction and characteristic of bipolar disorder [22]. In our study, *Gpr143* ^*−/y*^ mice strongly preferred sucrose solution (Figure 2), behaving similarly to the higher sucrose preference reported for mice receiving morphine [22]. The enhanced hedonic and reward responses in *Gpr143* ^*−/y*^ mice suggest that the loss of *Gpr143* gene may affect the functions of neural circuits that are involved in reward systems, in which the mesolimbic dopaminergic pathway plays an important role. For example, it was reported that, in the two‐bottle choice test, pimozide, an atypical D2R antagonist, decreased sucrose intake but increased water intake [43]. Similarly, optogenetic inhibition of D2R‐expressing neurons decreased motivation while activating the nucleus accumbens D2R‐expressing neurons increased cue‐induced motivational drive [44]. On the other hand, however, activation of D1R stimulates the expression of reward‐related behaviors, and activation of D2R stimulates the expression of aversion‐related behaviors. Since distinct brain regions and multiple neuron innervation have been implicated in distinct processes [45], the enhanced hedonic and reward responses might reflect dopaminergic as well as other unknown neurotransmitter dysregulation in *Gpr143* ^*−/y*^ mice.

In the tube test, *Gpr143* ^*−/y*^ mice exhibited significantly more pushes and resisted being pushed more than *Wt* mice, indicating greater aggression to achieve social dominance (Figure 4) [28, 30]. This is in line with other studies investigating the role of the D2R in social dominance. In one study, the knockdown of D2Rs in the dorsomedial prefrontal cortex, an area implicated in social hierarchy, led to reduced aggressive behavior and greater losses in the tube test, and D2R expression was significantly downregulated in the dorsal part of the medial prefrontal cortex in these lower rank mice [28]. Although neurotransmitter or modulator systems underlying the social hierarchy in a group are not limited to the DA nervous system, it is possible that altered D2R function could be causally related to the higher social dominance in *Gpr143* ^*−/y*^ mice. Indeed, increased aggressiveness to dominate a social hierarchy is a symptom of a mood or affective disorder (often during a manic or hypomanic phase) or a personality disorder.

Such behavioral phenotypes observed in *Gpr143* ^*−/y*^ mice are thought to reflect the function of brain regions where GPR143 is expressed; however, it is also possible that they result from functional changes in distant regions where GPR143 is not expressed. The microarray analysis in this study elucidated the global changes in gene expression in the *Gpr143* ^*−/y*^ mouse brain as compared to the Wt. We identified 17 downregulated and 20 upregulated genes in the forebrain of *Gpr143* ^*−/y*^ mice (Figure 5A and Table 2). The downregulated genes included, as might be expected, those related to Parkinson's disease (PD) (Vps13d), dopaminergic neuron differentiation (Nr2f1), regulation of DA receptor expression (Klf16), DA transporter (DAT) trafficking (Vav2), but also those linked to β‐catenin signaling (Tnks), synaptic proteins (Plekha5), and stimulus–response habits (Abl2). The upregulated genes included genes related to DA receptor signaling (Ctcf), D2R‐mediated ERK activation (Hbegf), as well as learning and memory (Wdr1, Anapc2) and neurotrophic factors (Hdgf). Although the microarray data require further validation, these findings suggest significant changes in genes that may be involved in serotonergic and/or dopaminergic transmission (Klf16, Vav2, Ctcf, Hbegf, Clathrin light chain B), neuronal differentiation (Nr2f1, Hdgf), drug addiction (Abl2) and synaptic plasticity (Plekha5, Wdr1, Anapc2). Some or all of these alterations may contribute to the behavioral phenotypes observed in *Gpr143* ^*−/y*^ mice.

GPR143 is widely expressed in the brain of mice [46], rats [47] and humans [48, 49]. GPR143‐immunoreactive or GPR143 mRNA‐positive cells are observed in the hippocampus, cerebral cortex, cerebellar cortex, striatum, substantia nigra, nucleus accumbens, ventral tegmental area, hypothalamic median eminence and supraoptic nucleus, nucleus tractus solitarii and caudal ventrolateral medulla and rostral ventrolateral medulla, medial habenular nucleus and olfactory bulb. As discussed above, GPR143 modifies dopaminergic transmission by coupling with D2Rs [7, 8, 9]. In the striatum and nucleus accumbens, some of the GPR143‐positive cells were surrounded by tyrosine hydroxylase (TH)‐immunoreactive fibers. Consistently, DOPA as well as DA is released upon selective stimulation of TH‐positive neurons in the nigrostriatal dopaminergic neurons [7]. On one hand, immunohistochemical analysis using anti‐DOPA and ‐DA antibodies or anti‐aromatic l‐amino acid decarboxylase (AADC) or ‐TH demonstrated that some neurons exhibited DOPA‐positive but DA‐negative and TH‐positive and AADC‐negative in the brain regions including the nucleus tractus solitarii, cerebral cortex and hypothalamus [50, 51]. Although GPR143 pathways involved are unknown at present [52], these findings support the notion that loss‐of‐function of GPR143 in these brain regions may be involved in the behavioral phenotypes such as impaired PPI (striatum, hippocampus, cortex) (Figure 1), sucrose preference (nucleus accumbens, hippocampus) (Figure 2), social dominance (cerebral cortex, nucleus accumbens) (Figure 3), depressive‐like behavior (hippocampus, amygdala, cerebral cortex, nucleus accumbens) (Figure 4). In adult *Gpr143* ^*−/y*^ mice, hippocampal neurogenesis was decreased during development and adulthood, and these mice showed exacerbated depression‐like behavior [34]. As hippocampal neurogenesis also plays a crucial role in learning and memory, this finding is also consistent with the phenotype of minor cognitive impairment of *Gpr143* ^*−/y*^ mice [34].

We identified gene clusters using ARCHS4 coexpression database in EnrichR (Figure 5B). The pathways involved include AKT/MEK/GSK/ERK, which have been implicated downstream of canonical GPCRs including adrenergic receptor alpha1 (ADRA1) and D2R. Indeed, this is consistent with our previous findings that GPR143, when coupled with ADRA1, D2RL, and D2RS modifies the downstream signaling of these GPCRs [7, 8, 9, 33]. IPA of the differentially expressed genes in the *Gpr143* ^*−/y*^ mice suggested DOPA to be the most significant upstream regulator related to GPR143 (Figure 5C). This finding is particularly interesting given that some of the actions of DOPA were absent in *Gpr143* ^*−/y*^ mice [7, 8, 9, 33, 34]. Several genes with altered expression have been implicated in schizophrenia and other mood disorders. For example, neuronal HB‐EGF has been found to be upregulated in schizophrenia, and similarly seen in our *Gpr143* ^*−/y*^ mice, and in these studies of schizophrenic patients, serum EGF levels were negatively correlated with cognition in patients with schizophrenia [53]. WDR1, involved in actin formation and auditory perception, has been shown to be differentially expressed in the dorsolateral prefrontal cortex of schizophrenic patients [54] and haplotype analyses have implicated the WDR1 locus in bipolar disorder as well [55]. Thus, the differential expression of molecules downstream of DOPA shows genetic changes both similar to schizophrenia and affective disorders like bipolar disorder.

Perhaps most intriguingly, the behavioral profile of the *Gpr143* ^*−/y*^ mice closely resembles those observed in a patient with a deletion of exons 2 through 8 of GPR143 [56]. This patient exhibited impulsivity, onset of schizophrenia during adolescence, intellectual impairment, and a chaotic social situation with a family history of substance abuse [56]. These findings are consistent with the expression pattern of GPR143, which is predominantly found in the primary targets of the mesolimbic and nigrostriatal dopamine systems, including the cerebral cortex, nucleus accumbens, ventromedial caudate putamen, and hippocampus [46, 47]. One limitation of the present study is that some of the original behavioral data were obtained over a decade ago, and it was impossible to trace all the previous records including birth dates and cage information. Although all animals were bred and maintained under standardized environmental conditions and were age‐matched at the time of testing, we could not completely exclude subtle cage or background effects. This limitation should be considered when interpreting the present findings.

In conclusion, GPR143 gene deficiency in mice leads to behaviors that model features from a mix of phenotypes, including impaired PPI and a mild cognitive deficiency suggestive of schizophrenia and depression, excessive reward seeking, and social aggressiveness suggestive of mood or affective disorders. Further studies will be required to elucidate the detailed pathogenic mechanisms, and whether compensating for GPR143 may form the basis of a therapeutic strategy.

## Author Contributions

Y.G., D.M., and E.Y.S. conceptualized, designed, interpreted results, and wrote the manuscript. H.N., M.K., K.S., and J.K. performed behavioral analysis; Y.K., M.N.V., and M.A. performed microarray and pathway analysis. All authors have read and approved the final manuscript.

## Funding

This work was supported by Scientific Research (B) (General) (Grant 21H02673 to Y.G.); Scientific Research (C) (General) (Grant 20K07069 to D.M.); and Foundation of Strategic Research Projects in Private Universities, from the Ministry of Education, Culture, Sport, Science, and Technology (MEXT) of Japan; the Japanese SRF Grant for Biomedical Research (Grants 1516 and 2021T001 to Y.G., 2021G010 to D.M.); the JPMH KAKENHI (Grant 22KC1005); Scientific Research (C) (General) (Grant 20K07069 to D.M.), Uehara Memorial Foundation (Grant 201810115 to D.M.) and the Fund for Creation of Innovation Centers for Advanced Interdisciplinary Research Areas Program in the Project for Developing Innovation Systems from MEXT (Grant 42890001 to Y.G.); Takeda Science Foundation (to D.M.), and AMED (JP22ek0109431 to Y.G.). M.N.V. was supported by a Merkin Foundation grant from the UCSD.

## Ethics Statement

The study was conducted according to the protocol approved by the President of Yokohama City University after reviewed by the Institutional Animal Care and Use Committee (Approval No. F‐A‐14‐046). In all studies, the authors followed the guidelines for the use and care of laboratory animals of Yokohama City University.

## Consent

The authors have nothing to report.

## Conflicts of Interest

The authors declare no conflicts of interest.

## Supporting information


**Data S1:** npr270080‐sup‐0001‐Supinfo.docx.

**Table S1:** npr270080‐sup‐0002‐TableS1.xlsx.

**Table S2:** npr270080‐sup‐0003‐TableS2.xlsx.

## Data Availability

GEO accession number for micro array analysis was GSE157828. The study data is available as Supporting Information.
