# Supplementary material for: Disruption of the l‐DOPA Receptor Gpr143/OA1‐Gene in Mice Creates a Unique Mixed Psychosis‐Like Phenotype
Source: Neuropsychopharmacol Rep. 2026 Jan 9;46(1):e70080. doi: 10.1002/npr2.70080 (PMC12784213; doi:10.1002/npr2.70080)
Supplement: Supplementary file 1 — Data S1: npr270080‐sup‐0001‐Supinfo.docx. [file NPR2-46-e70080-s003.docx]

**Supplementary Material**

**Disruption of the L-DOPA receptor *Gpr143/OA1*-gene in mice creates a unique mixed psychiatric phenotype that emulates components of schizoaffective disorder**

Yoshio Goshima, MD&PhD^1,7*^, Hiromi Okatsu^1^, Haruko Nakamura, MD&PhD ^2^, Motokazu Koga, MD&PhD ^3^, Junka Koyama, MD^1^, Yayoi Kimura, PhD^4^, Maya N. Vasishth, MSc^5^, Evan Y. Snyder, MD&PhD ^5,6^, Masashi Asai, PhD^7^, Kenta Sakai, PhD^7^, Daiki Masukawa, PhD^1,*^

^1^Department of Molecular Pharmacology and Neurobiology, Yokohama City University Graduate School of Medicine, 3-9 Fukuura, Kanazawa-ku, Yokohama, 236-0004, Japan

^2^Department of Neurology, Yokohama City University Graduate School of Medicine, 3-9 Fukuura, Kanazawa-ku, Yokohama, 236-0004, Japan

^3^Department of anesthesiology, Kanagawa Cancer Center, 2-3-2 Nakao Asahi-ku, Yokohama, 241-8515, Japan

^4^Advanced Medical Research Center, Yokohama City University, Yokohama 236-0004, Japan

^5^Sanford Burnham Prebys (SBP) Medical Discovery Institute, La Jolla, CA 92037, USA

^6^Biomedical Sciences Graduate Program, University of California-San Diego, La Jolla, CA 92037, USA

^7^Department of Kampo Pharmacy, Yokohama University of Pharmacy, Yokohama 245-0066, Japan

^8^ Environmental Health and Prevention Research Unit, Yokohama University of Pharmacy, 601 Matano, Totsuka, Yokohama 245-0066, Japan

*Correspondence: E-mail: [goshima@yokohama-cu.ac.jp](mailto:goshima@yokohama-cu.ac.jp), masukawa@yokohama-cu.ac.jp

**Contents**

**Supplementary Figures and Legends**

**Fig. S1. Open field test.** No significant differences were observed between *Wt* and *Gpr143-/y* mice in total moving distance **(A)**, moving time per minute **(B)**, or total time spent in the center **(C)**. Data are shown as mean ± SEM (n = 8).

**A**

**B**

**Fig. S2. No significant differences in motor function between *Wt* and *Gpr143^-/y^* mice.**

Neither in the rotarod test **(A)** nor in the **(B)** balance beam test (latency to traverse) were significant differences observed between *Wt* and *Gpr143^-/y^* mice. Data are shown as mean ± SEM (n = 8).

**Fig. S3. Olfactory habituation/dishabituation test.** No significant differences in olfactory function were observed between *Wt* and *Gpr143^-/y^* mice. W, water; V, vanilla; A, bitter almond. Data are shown as mean ± SEM (n = 8).

**Fig. S4. Light–dark exploration test.** No significant differences were observed in time spend in the light compartment **(A)**, time spent in the dark compartment **(B)**, or the number of entries into the dark compartment **(C)** between *Wt* and *Gpr143^-/y^* mice. Data are shown as mean ± SEM (n = 8).

**Fig. S5. Contextual/cued fear conditioning test.** No significant differences in freezing time were observed during base line conditioning **(A)** or place **(B)**/sound **(C)** conditioning between *Wt* and *Gpr143^−/y^* mice. Data are shown as mean ± SEM (n = 8).

**Fig. S6. Elevated plus maze test.** No significant differences were observed in the mean time spent on **(A)** and the mean distance traveled **(B)** within the open arm or closed arm between *Wt* and *Gpr143^-/y^* mice. Data are shown as mean ± SEM (n = 8).

**Fig. S7. Social interaction test.** No significant differences were observed in the time spent exploring familiar and novel mouse between *Wt* and *Gpr143^-/y^* mice. Data are shown as mean ± SEM (n = 8 or 7).
